# Short-course versus long-course neoadjuvant chemoradiotherapy in patients with rectal cancer: long-term results of a randomized controlled trial

**DOI:** 10.1007/s00384-025-04901-1

**Published:** 2025-05-14

**Authors:** Mahdi Aghili, Mohammad-Mahdi Aghaei, Romina Abyaneh, Mohammad Babaei, Farshid Farhan, Marzieh Lashkari, Borna Farazmand, Kasra Kolahdouzan, Guglielmo Niccolò Piozzi, Felipe Counago, Reza Ghalehtaki

**Affiliations:** 1 Radiation Oncology Research Center, Cancer Research Institute, IKHC, Tehran University of Medical Sciences, Tehran, Iran; 2 Department of Radiation Oncology, Cancer Institute, IKHC, Tehran University of Medical Sciences, Tehran, Iran; 3 Department of Colorectal Surgery, Portsmouth Hospitals University NHS Trust, Portsmouth, UK; 4 Department of Medicine, Faculty of Medicine, Health and Sport Sciences, Universidad Europea de Madrid, Madrid, Spain; 5 GenesisCare, Madrid, Spain; 6 Hospital Universitario San Francisco de Asís, Madrid, Spain; 7 Hospital Universitario La Milagrosa, Madrid, Spain

**Keywords:** Rectal cancer, Neoadjuvant chemoradiotherapy, Short-course radiotherapy, Long-course radiotherapy

## Abstract

**Background and purpose:**

Short-course radiotherapy (SCRT) and long-course radiotherapy (LCRT) are the primary neoadjuvant radiotherapy schedules for locally advanced rectal cancer. Recent research has questioned the efficacy of SCRT. This study presents an updated analysis of our previous research, extending the follow-up to evaluate 5-year outcomes by comparing the long-term results of these two strategies.

**Materials and methods:**

This randomized controlled trial compared SCRT and LCRT in locally advanced middle or high rectal adenocarcinoma. The SCRT group received 25 Gy/5 fractions over 1 week plus CAPOX, while the LCRT group received 50–50.4 Gy/25–28 fractions over 5–5.5 weeks plus capecitabine. All patients received consolidation chemotherapy and then underwent delayed surgery after 8 weeks or more post-radiotherapy. The endpoints of this updated analysis include overall survival (OS),disease-free survival (DFS), locoregional recurrence (LR) and distant metastasis (DM).

**Results:**

Ninety-nine cases (45 LCRT, 54 SCRT) were followed for a median of 4.7 years. Five-year OS rates were 77.3% for LCRT vs. 65.6% for SCRT group (*P* = 0.4). The 5-year DFS rates were 69.6% for LCRT vs. 54.9% for SCRT (*P* = 0.07). Cox regression indicated no prominent difference between the two groups regarding OS, LR, or DM. Subgroup analysis demonstrated a significantly better DFS with LCRT compared to SCRT in male patients ([HR] = 2.48, 95%CI: 1.04–5.93, *P* = 0.03), patients under the age of 60 (HR = 3.19, 95%CI: 1.03–9.92, *P* = 0.04), and cT4 patients (HR not calculated: no events in LCRT group, *P* = 0.004).

**Conclusion:**

DFS showed a trend in favor of the LCRT group, with LCRT being significantly superior among men, patients under 60, and cT4 stage. Despite being intensified, SCRT failed to achieve long-term outcomes comparable to LCRT. Further research is needed to compare these two approaches in the context of total neoadjuvant treatment.

**Trial registration data:**

IRCT2017110424266N3 (Registration date: 2017–11-12). https://irct.behdasht.gov.ir/trial/20526.

**Supplementary Information:**

The online version contains supplementary material available at 10.1007/s00384-025-04901-1.

## Introduction

Colorectal cancer (CRC) is the third most common cancer worldwide, with rectal cancer accounting for about one-third of all cases. Similarly, in Iran, CRC ranks third in men and fourth in women [1, 2]. A notable global rise in colorectal cancer is observed in individuals under 50, imposing a significant burden on younger populations, particularly in high-income countries. Contributing factors may include Western-style diets, obesity, and physical inactivity [3].

Historically, standard treatment for locally advanced rectal cancer (LARC) involves a combination of neoadjuvant radiotherapy, either short-course (SCRT) or long-course (LCRT), with or without chemotherapy, followed by surgery. LCRT, commonly used in the U.S. and several European countries, delivers 45–54 Gy in 25–28 fractions along with chemotherapy, usually 5-fluorouracil (5 FU) or its derivatives. Surgery is performed 6–8 weeks later [4]. In contrast, SCRT, more commonly used in Scandinavia and Poland, involves a 25 Gy dose in 5 fractions without chemotherapy, followed by surgery within one week of completing radiotherapy [5].

Conventional LCRT with chemotherapy has some downsides. It results in an elongated treatment period and increases the time between diagnosis and surgery. Furthermore, it is an expensive treatment, and patients may experience long waiting lists. Recent studies have highlighted several approaches to improve the outcomes of LCRT, including radiotherapy intensification, concurrent chemotherapy intensification, and total neoadjuvant therapy (TNT). However, further long-term studies are necessary to validate these findings [6, 7].

SCRT is characterized by a shorter treatment time that leads to a shorter waiting time, taking only one-fifth of the time required for LCRT. In most studies, SCRT without chemotherapy has shown lower pathological complete response (pCR) rates than conventional LCRT [8]. Moreover, there is no existing consensus on which SCRT regimen would produce the highest response rate [9, 10]. However, it has been shown that SCRT plus concurrent chemotherapy followed by consolidation chemotherapy and postponed surgery is viable and well-tolerated without notable toxicity and is linked to encouraging pCR rates [11]. The RAPIDO trial compared SCRT and LCRT, revealing that SCRT achieved a higher pCR rate [12]. However, long-term follow-up indicated a higher rate of local recurrence with SCRT. These variations among different studies’ findings suggest the need for further investigations and a more careful patient selection strategy to determine which patients would benefit more from SCRT versus LCRT [13].

In recent years, TNT has been proposed, which includes short-course or long-course radiotherapy and induction or consolidation full-dose chemotherapy before surgery. This method is supposed to have higher patient compliance as there is no need for further chemotherapy after surgery. Additional research must be done to compare short-course and long-course radiotherapy approaches in TNT [14–16].

Few studies directly compare SCRT and LCRT with the same chemotherapy regimens and equivalent intervals from the end of radiotherapy to surgery. Previous research has shown that pCR rates and long-term follow-up outcomes can diverge, and our previous report of this trial demonstrated an insignificantly higher pCR rate in the SCRT group [17]. To address the gap regarding the association of early outcomes like pCR and survival outcomes, we compared long-term follow-up results including overall survival (OS), disease-free survival (DFS), locoregional recurrence (LR), and distant metastasis (DM) by adding more patients to enhance the robustness of our findings compared to previous studies.

## Materials and methods

### Study design and participant

This report presents the long-term outcomes of the previous trial version, with complete accrual, and the methodology has been thoroughly explained [18]. Recruitment of patients for this randomized controlled clinical trial was carried out between April 2016 and April 2019 at the radiation oncology department of the Cancer Institute of Iran. The trial adhered to the CONSORT (Consolidated Standards of Reporting Trials) guidelines to ensure transparent and comprehensive reporting. The study included patients with rectal adenocarcinoma confirmed by histological diagnosis, located at least 5 cm from the anal verge with the epicenter of the tumor below the line that connects sacral promontory and pubic symphysis. Patients with cT3-4 stage and/or node-positive status were eligible for the study, as determined by magnetic resonance imaging (MRI) or endoscopic ultrasound (EUS). Patients were excluded from the study if they had a history of distant metastasis, an Eastern Cooperative Oncology Group performance status above 1, were deemed inoperable or unable to tolerate chemotherapy, had a recurrence following prior surgery, or had familial adenomatous polyposis. Additionally, individuals with a history of a second primary malignancy were not included.

### Pre-treatment assessment

**Fig. 1:**
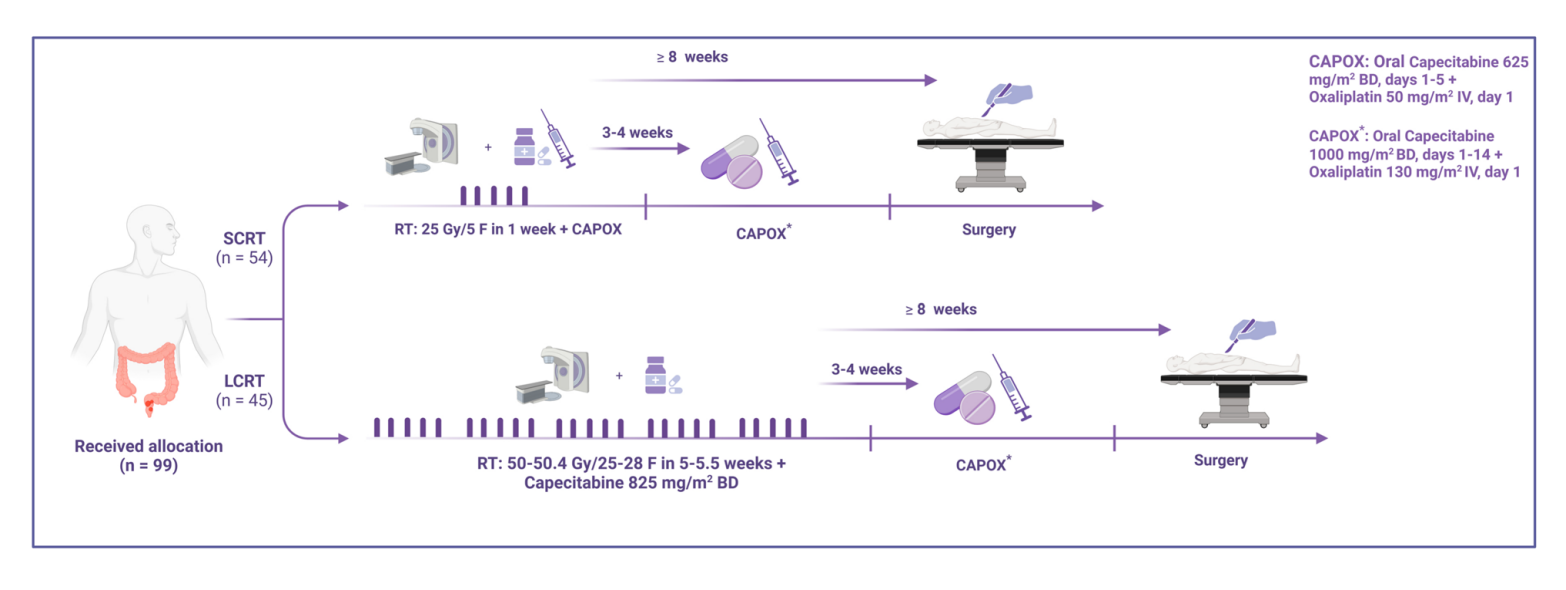
Trial design and treatment protocols. Legend: The SCRT regimen involved 3D-conformal radiotherapy (3D-CRT) delivering 25 Gy in five fractions over one week, combined with concurrent CAPOX chemotherapy (capecitabine 625 mg/m^2^ twice daily, days 1–5, and oxaliplatin 50 mg/m^2^ IV on day 1). The LCRT regimen included 3D-CRT delivering 50–50.4 Gy in 25–28 fractions over 5–5.5 weeks, with concurrent capecitabine 825 mg/m^2^ twice daily. Both groups underwent delayed surgery (at least 8 weeks after radiotherapy) and preoperative CAPOX chemotherapy (capecitabine 1000 mg/m^2^ twice daily, days 1–14, and oxaliplatin 130 mg/m^2^ IV on day 1), starting 3–4 weeks post-radiotherapy

The pre-treatment assessments involved imaging techniques, including pelvic MRI and/or EUS and thoracoabdominal CT scan, as well as laboratory tests such as complete blood count, liver and renal function tests, and serum carcinoembryonic antigen (CEA). Eligible patients were required to give informed consent following completion of the staging assessment and prior to randomization. Those consenting to participate were randomly allocated to either the SCRT or LCRT treatment group on a 1:1 basis, with the patients in each group matched based on the stage of their cancer. The randomization was performed using the permuted block method. As the study intervention was specific, the participants could not be blinded to their respective group assignments. To reduce patient dropout and withdrawal, investigators contacted participants through regular phone follow-ups. Weekly physical exams and laboratory tests were conducted after the study began to track any post-treatment complications. Subsequent assessments were conducted four times a year during the first two years, biannually for the following five years, and annually thereafter. The treatment protocol for both groups is illustrated in Fig. 1. The SCRT regimen involved three-dimensional conformal radiotherapy (3D-CRT) with a total dose of 25 Gy delivered in five fractions over one week, combined with concurrent CAPOX chemotherapy (capecitabine at 625 mg/m^2^ twice daily from days 1 to 5, plus oxaliplatin with a dose of 50 mg/m^2^ administered intravenously on day 1 only). On the other hand, the patients in the LCRT group received 3D-CRT at a dose of 50–50.4 Gy in 25–28 fractions over 5 to 5.5 weeks, along with concurrent capecitabine 825 mg/m2 twice daily. Radiotherapy target delineation followed international guidelines established by Valentini et al. [19]. All participants received capecitabine tablets, which were supplied by Actero Middle East Company, also known as Actero Pharma, in Tehran, Iran. Patients in both treatment groups also received pre-operative chemotherapy with CAPOX (oral capecitabine 1000 mg/m^2^ twice a day, on days 1–14 plus oxaliplatin 130 mg/m^2^ IV on day 1), starting 3 to 4 weeks after completing radiotherapy. Delayed surgery was performed in both groups, scheduled no earlier than eight weeks after the completion of radiotherapy, either in the surgical oncology or colorectal surgery department. The administration of adjuvant chemotherapy depended on the treating physician's decision, considering the patient’s risk, treatment response, primary stage, and performance status.

### Outcome assessment

This study primarily analyzed 5-year follow-up outcomes as part of secondary endpoint assessments and post-hoc analyses. The primary endpoint of the original trial was acute toxicity, as reported in our previous publication. This report focuses on secondary endpoints, including OS, DFS (defined as survival without local failure or distant metastasis), LR, and DM. Additionally, post-hoc subgroup analyses of DFS were performed based on sex, age (cutoff of 60 years), clinical T and N stages, and pCR status to explore potential differences between SCRT and LCRT.

### Statistical analysis

The primary analysis was performed on an intention-to-treat (ITT) basis, as specified in the original trial protocol. Similarly, in the current study, we have included all patients who initiated the allocated treatment in the analysis. Continuous variables were reported as mean ± standard deviation (SD), while categorical variables were presented as frequencies and percentages. Group comparisons were conducted using the Student's t-test and chi-square test, as appropriate. OS and DFS were specified as secondary endpoints in the original trial design. We utilized the Kaplan–Meier method to construct analysis graphs for comparing the SCRT and LCRT groups, and cumulative hazard curves for LR and DM were generated. To compare the LCRT and SCRT groups, *p*-values were calculated using the log-rank test. The Cox proportional hazards model was used to estimate the hazard ratios (HRs) and 95 percent confidence intervals (95% CIs), with the LCRT group taken as the reference variable. In addition to the pre-specified secondary endpoints, post-hoc analyses were conducted to explore potential factors influencing DFS including sex, age by the cutoff of 60 years, clinical T and N, and pCR. Epidemiological data indicates that the median age for rectal cancer diagnosis has recently dropped to 63 years. Additionally, colorectal cancer stands as a significant leading cause of cancer-related mortality among individuals aged 60 to 79 [20, 21]. Consequently, we aimed to establish 60 years as a cutoff to compare the effects of SCRT versus LCRT approaches. The DFS results of LCRT and SCRT groups were compared in those subgroups, and hazard ratios and *p*-values were calculated using the Cox proportional hazards model and Wald’s test or log-rank test. Statistical analysis was performed using R software (version 4.3) using survival, survminer, and forest ploter packages.

### Ethical considerations

All participants provided written informed consent prior to study enrollment. The Ethics Committee of Tehran University of Medical Sciences approved this study, with the Ethics Code being IR.TUMS.VCR.REC.1396.3475 for the original study and IR.TUMS.IKHC.REC.1401.398 for the release of updated analysis. The study is registered in the Iranian Registry of Clinical Trials (IRCT) under the registration number IRCT2017110424266N3 (registration date: 2017–11–12). Additionally, this study was reviewed by an Institutional Review Board (IRB), with IRB approval number 1401–4–417–63773 for the long-term update.

## Results

**Fig. 2:**
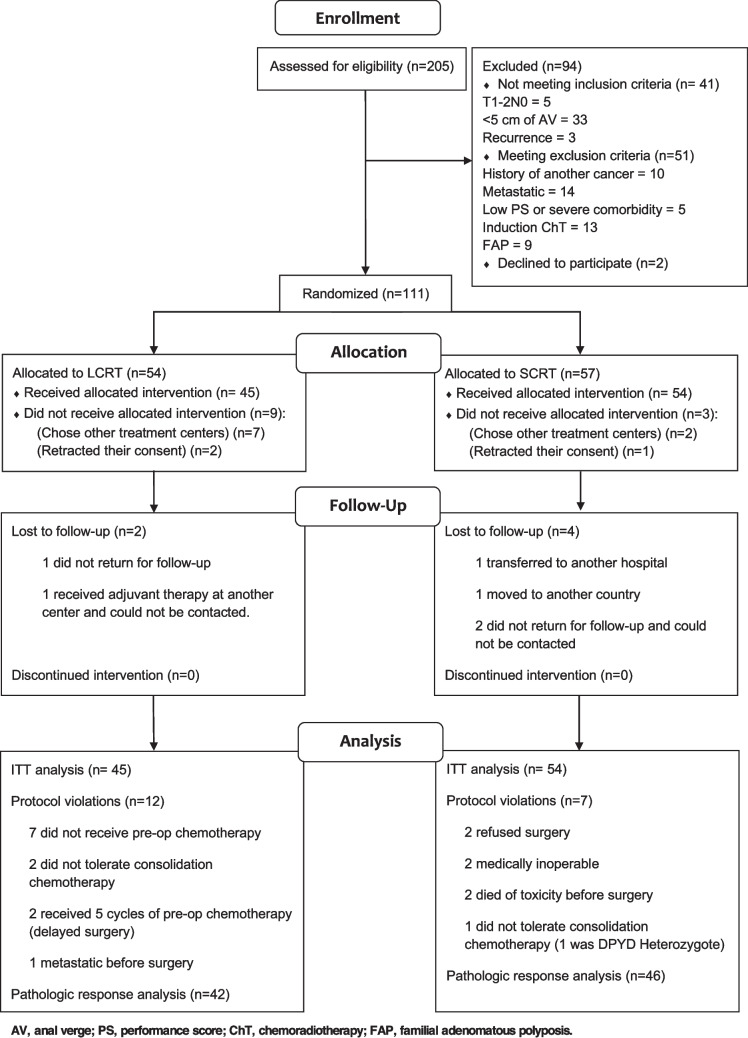
CONSORT flow diagram of the study. Legend: CONSORT diagram outlining the patient enrollment and analysis process. A total of 205 patients were assessed for eligibility, with 94 excluded. Then, 111 patients were randomized: 54 to the LCRT group and 57 to the SCRT group. In the LCRT group, nine patients did not receive the allocated intervention due to choosing other treatment centers (*n* = 7) or retracting consent (*n* = 2). In the SCRT group, three patients did not receive the allocated intervention due to choosing other treatment centers (*n* = 2) or retracting consent (*n* = 1). Two patients were lost to follow-up in the LCRT group, while four were lost to follow-up in the SCRT group. Protocol violations occurred in both groups (LCRT: *n* = 12, SCRT: *n* = 7). All patients who started the allocated intervention were included in the analysis according to the intention-to-treat (ITT) principle

Initially, 205 patients were recruited by April 2019. According to the CONSORT flow diagram (Fig. 2), 111 patients were assigned to either LCRT or SCRT based on inclusion and exclusion criteria. Ultimately, 99 patients received the treatments and were included in the analysis. Two patients in the LCRT group and four in the SCRT group were lost to follow-up, but no patients discontinued treatment. Efforts to maintain comparable group sizes were made; however, 45 patients were in the LCRT group and 54 in the SCRT group due to some retracting their consent or opting for treatment at other centers.

### Patient characteristics

Patients’ characteristics and surgical data in both treatment groups, as displayed in Table 1, did not indicate any significant differences between the two groups. The mean age ± SD for the LCRT and SCRT groups were 54.09 ± 11.61 and 57.78 ± 11.29, respectively (*P* = 0.11). In both groups, males were the majority of patients, with 60% in the LCRT group and 63% in the SCRT group (*P* = 0.92). Following neoadjuvant treatment, 9 patients (7 SCRT, 2 LCRT, *p* = 0.14) did not undergo surgery. The reasons were unknown for 2 cases (1 SCRT, 1 LCRT). However, in the SCRT group, 2 patients refused surgery, 2 died during or shortly after neoadjuvant therapy, and 2 were medically inoperable. In the LCRT group, one developed metastatic disease before surgery.

**Table 1:** Characteristics of patients in the study, including baseline characteristics and surgery data

	Long-course	Short-course	*p*-value
Age			0.28
Under 60 years	29 (64.4)	29 (53.7)	
60 years and above	16 (35.6)	25 (46.3)	
Sex			0.91
Female	18 (40)	21 (38.9)	
Male	27 (60)	33 (61.1)	
Distance from anal verge (cm)			0.45
Less than 10	26 (59)	37 (68.5)	
10 or more	18 (41)	17 (31.5)	
Histological grade (*n* = 75)			0.65
1	13 (44.8)	17 (37)	
2	12 (41.4)	19 (41.3)	
3	4 (13.8)	10 (21.7)	
Clinical T status (*n* = 98)			0.69
Lower than 4	37 (84.1)	48 (88.9)	
4	7 (15.9)	6 (11.1)	
Clinical N status (*n* = 99)			0.21
0–1	29 (64.4)	42 (77.8)	
2	16 (35.6)	12 (22.2)	
Surgery performed (*n* = 99)			0.142
Yes	43 (95.6)	47 (87)	
No	2 (4.4)	7 (13)	
Pathological TNM stage (*n* = 78)			0.622
0	10 (24.4)	12 (32.4)	
1	14 (34.1)	8 (23.5)	
2	13 (31.7)	12 (32.4)	
3	4 (9.8)	5 (13.5)	
pCR (*n* = 89)	10 (23.8)	12 (25.5)	0.851
TRG (*n* = 87)			0.371
0–1	20 (50)	19 (40.5)	
2–3	20 (50)	28 (59.5)	
R0 resection	38 (100)	35 (97.2)	0.48
Resected Nodes	8.05 ± 8.21	9.39 ± 7.22	0.46
Perineural Invasion (*n* = 59)	3 (8.1)	7 (21.9)	0.17
Lymphovascular invasion (*n* = 70)	12 (32.4)	7 (21.2)	0.43

### Treatment timing and consolidative chemotherapy

The time intervals between treatment phases were analyzed for the entire cohort and compared between the SCRT and LCRT groups. From the start of radiation to surgery, the median time was 12.5 weeks (IQR: 10–18) in the whole population, with significant differences between the SCRT (median: 10.5 weeks, IQR: 8.25–12) and LCRT groups (median: 18 weeks, IQR:15–25; *P* < 0.001). Similarly, the interval from the end of radiation to surgery was shorter in the SCRT group (median: 10 weeks, IQR: 8–11) compared to the LCRT group (median: 12 weeks, IQR: 10–19; *P* < 0.001), with a median of 11 weeks (IQR: 9–14) overall. The time from the end of chemotherapy to surgery also varied, with a median of 7 weeks (IQR: 5–9) in the entire cohort, though the SCRT group had a shorter median (6 weeks, IQR: 4.25–8) than the LCRT group (7 weeks, IQR: 6–11; *P* = 0.025).

Six patients were unable to receive at least one cycle of consolidation chemotherapy. One patient developed an infection during the preoperative period, another experienced metastatic progression before surgery and discontinued treatment, and one was medically unfit for surgery and did not return for further therapy. Additionally, one patient did not receive chemotherapy due to poor performance status during the preoperative period. The remaining two patients did not return for consolidation chemotherapy and instead proceeded directly to surgery. Specifically, 89 cases (89.9%) received only one cycle of consolidation chemotherapy, 2 cases (2%) received two cycles of consolidation chemotherapy, 1 case (1%) received three cycles, and 1 case (1%) received five cycles, and 6 cases (6.1%) did not receive even one cycle of consolidation chemotherapy. The median duration between radiotherapy completion and consolidation chemotherapy initiation was 3 weeks (IQR: 2–4), with no significant difference between the SCRT and LCRT groups and most of the patients completed the planned cycles without major deviations.

### Long-term follow-up results in short-course and long-course chemoradiotherapy

**Fig. 3:**
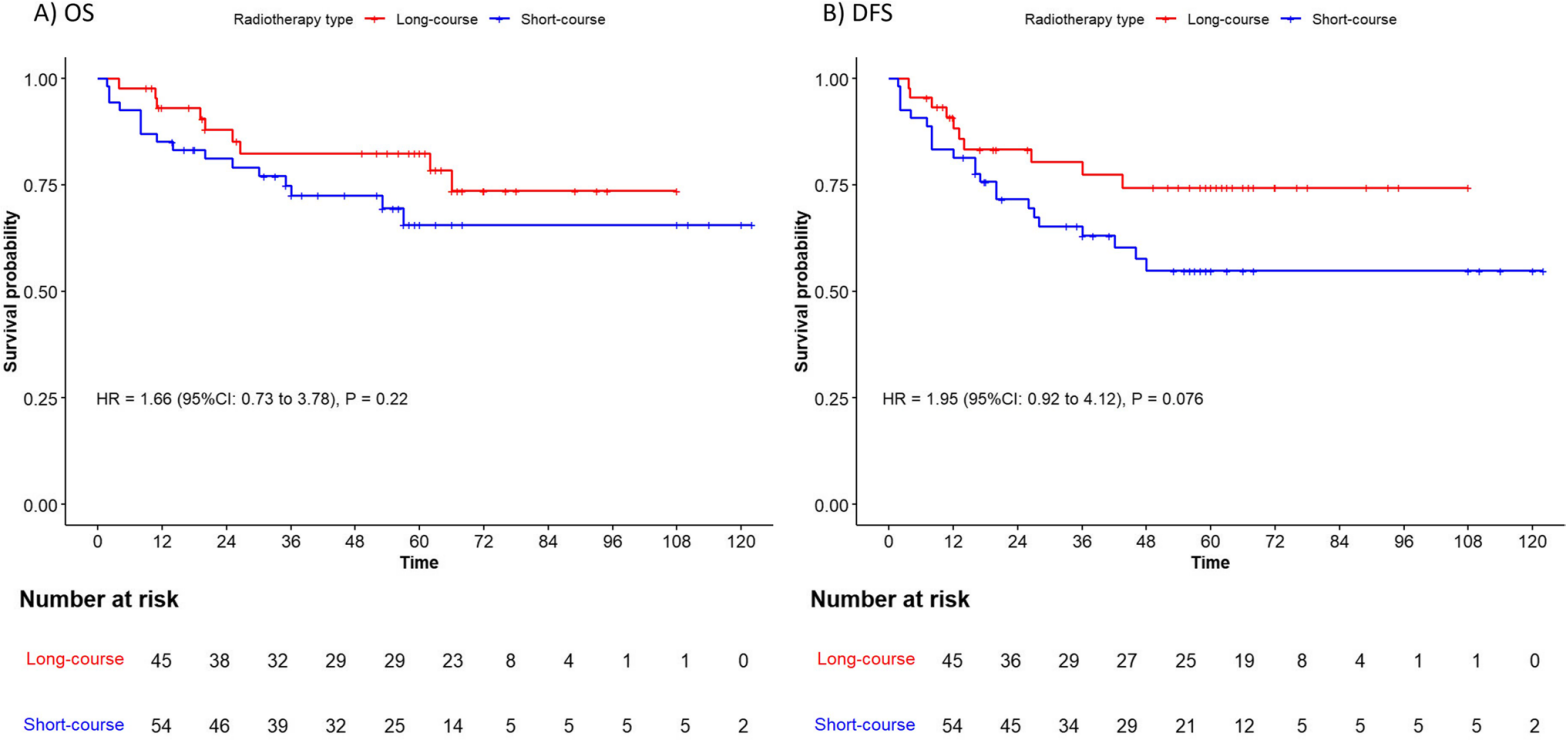
Kaplan–Meier curves for overall survival and disease-free survival: LCRT vs. SCRT. Legend: Kaplan–Meier plots showing (**A**) overall survival (OS) and (**B**) disease-free survival (DFS) for patients treated with the LCRT and SCRT approaches. Hazard ratios (HR) for the SCRT group were calculated using univariate Cox regression analysis, and *P*-values were determined using the log-rank test

**Fig. 4:**
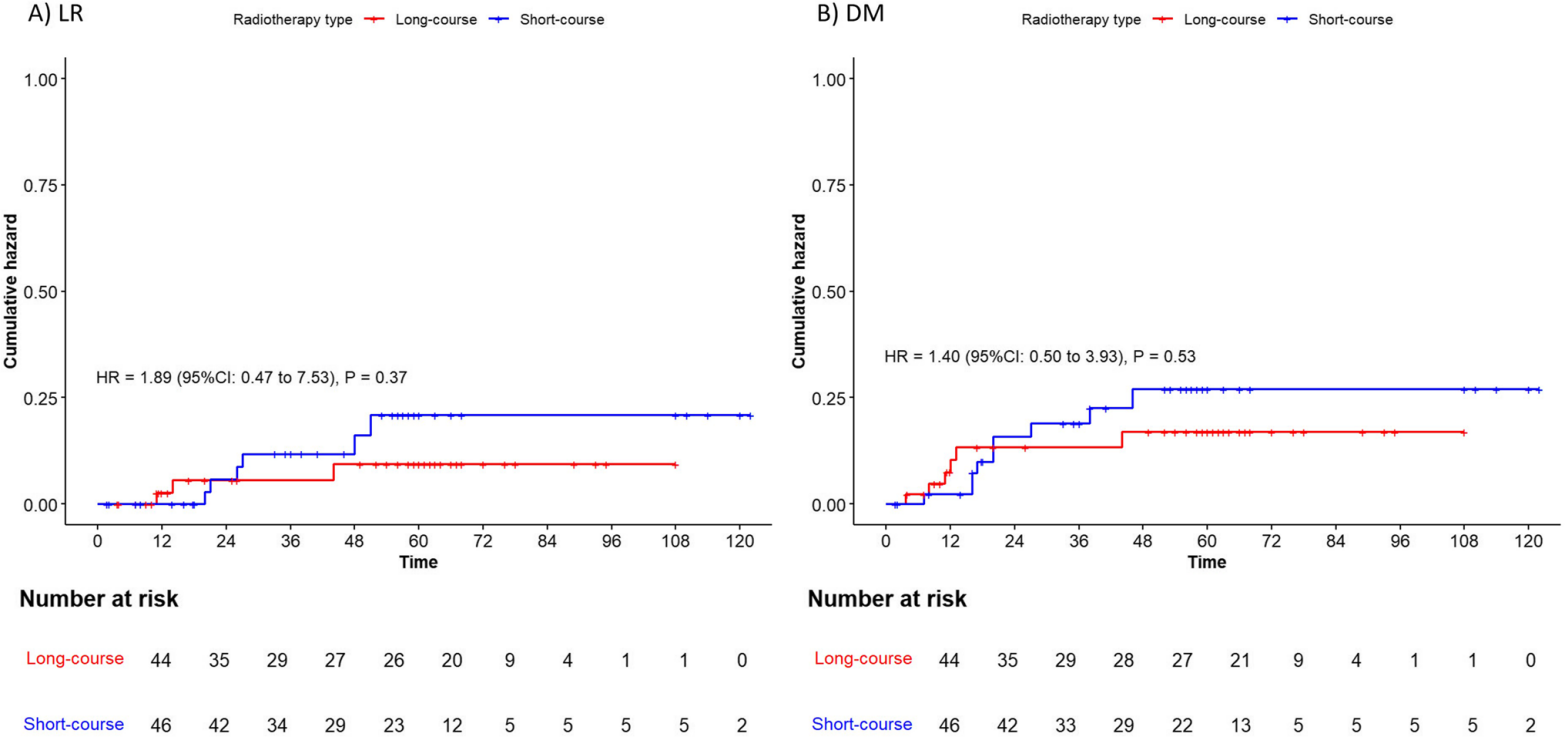
Cumulative hazard of local recurrence and distant metastasis: LCRT vs. SCRT. Legend: Cumulative hazard plots illustrating (**A**) locoregional recurrence (LR) and (**B**) distant metastasis (DM) for patients treated with the LCRT and SCRT approaches. Hazard ratios (HR) for the SCRT group were calculated using Cox regression analysis, and *P*-values were determined using the log-rank test

Kaplan–Meier plots comparing the long-term follow-up results of the LCRT and SCRT groups are shown in Figs. 3 and 4*.* Kaplan–Meier plots of the OS (Fig. 3A), LR (Fig. 4A), and DM (Fig. 4B) over the follow-up period were not significantly different between the SCRT and LCRT groups. However, patients receiving LCRT treatment had a trend toward higher DFS rates than the SCRT group over the follow-up period (HR = 1.95, 95% CI: 0.92–4.12, *P* = 0.076, Fig. 3B). As shown in Table 2, five-year OS rates were higher in the LCRT group compared to the SCRT group; however, the difference did not reach statistical significance (77.3% vs. 65.6%, *P* = 0.4). Additionally, a trend towards higher 5-year DFS rates was observed in the LCRT group compared to the SCRT group (69.6% vs. 54.9%, *P* = 0.07).

**Table 2:** Comparison of 5-year OS and DFS rates between the LCRT and SCRT groups

	LCRT	SCRT	*p*-value
5-year overall survival rate	77.3% (95 CI: 65.3%−91.6%)	65.6% (95 CI: 52.6%−81.8%)	0.4
5-year disease-free survival rate	69.6% (95 CI: 56.5%−85.7%)	54.9% (95 CI: 42.1%−71.4%)	0.07

**Fig. 5:**
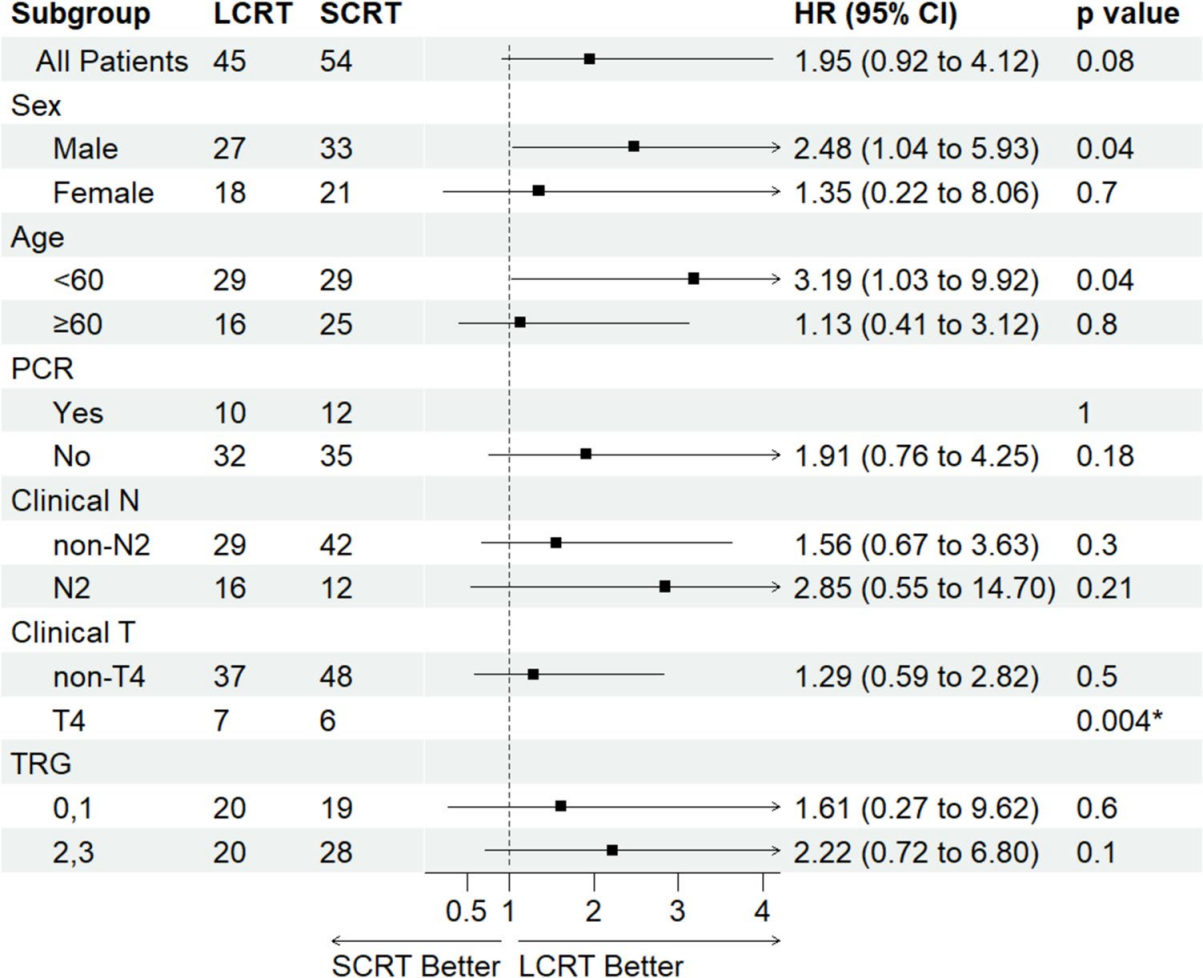
Forest plot of subgroup analysis for disease-free survival (DFS). Legend: Forest plot of subgroup analysis for disease-free survival (DFS) using Cox regression. Subgroups analyzed include sex, age, pathological complete response (pCR), clinical N stage, clinical T stage, and tumor regression grade (TRG). *P* value calculated by Wald’s test. However, in the cT4 subgroup, due to the loss of events in the LCRT group, Cox regression could not be performed, and the *p*-value was calculated usingthe log-rank method. The hazard ratio (HR) for the PCR (Yes) subgroup could not be estimated due to no events occurring in the LCRT arm

Patients were further stratified into subgroups based on sex, age by 60 years cutoff, pCR status, clinical N and T status (AJCC 7 th edition), and tumor regression grade (TRG, based on the modified Ryan system [22]. The DFS results of LCRT and SCRT groups were compared in those subgroups, and hazard ratios and *p* values were calculated. No significant difference was seen in the DFS rate between the SCRT and LCRT groups in each pCR, TRG, and clinical N status subgroup. In contrast, as shown in Figure S1 (in the supplementary section), males who received SCRT treatment had a significantly lower DFS rate than males who received LCRT treatment (HR = 2.48, 95% CI: 1.04–5.93, *P* = 0.03). As shown in Figure S2, SCRT had a significantly lower DFS rate in patients under the age of 60 (HR = 3.19, 95% CI: 1.03–9.92, *P* = 0.04) and also in clinical T4 patients, as shown in Figure S3. (HR could not be calculated due to the absence of events in the LCRT group, *p*-value by log-rank = 0.004). As shown in Figure S4, patients with a TRG of 0–1 (HR = 1.61, 95% CI: 0.27–9.62, *P* = 0.6) or a TRG of 2–3 (HR = 2.22, 95% CI: 0.72–6.8, *P* = 0.2) did not show any significant difference in DFS between LCRT and SCRT groups. The forest plot of the subgroup analysis is shown in Fig. 5.

## Discussion

While the original study primarily assessed acute toxicity, this analysis focuses on long-term oncologic outcomes as secondary endpoints. Additionally, post-hoc subgroup analyses were performed to explore potential differences in DFS, though these findings should be interpreted with caution as they were not pre-specified. Long-term follow-up results showed a remarkable trend favoring the LCRT approach over SCRT regarding DFS rates, though the difference did not reach statistical significance (*P* = 0.076). However, in male patients, those with a cT4 stage, and patients under 60, the DFS rate was significantly better in the LCRT group.

Similar to our previous report, we did not find any significant difference in pCR rates between the two groups [17]. However, pCR is often associated with favorable prognosis at an individual level, and in retrospective studies, its role as a surrogate for long-term survival outcomes remains uncertain in clinical trials, as discussed by Fokas et al. [23] and highlighted in the RAPIDO trial [12].

Concern regarding the durability of SCRT was raised after Dijkstra et al. released the five-year follow-up results of the RAPIDO trial. The long-term results indicated that the rate of locoregional recurrence was higher in the SCRT when used in the context of a TNT approach compared to the conventional non-TNT long-course chemoradiotherapy (10.6% vs. 6%). Additionally, patients receiving SCRT with locoregional failure received more often 3D-CRT compared with those in the LCRT group (*P* = 0.029) [13]. All patients in our study received 3D-CRT, which may partly explain the trend favoring LCRT for long-term survival, as intensity-modulated techniques were not used. Although our cohort was generally lower-risk than the RAPIDO trial population, which included high-risk patients and a subset treated with intensity-modulated radiation therapy (IMRT), we reached somewhat similar findings.

A recent study from Memorial Sloan Kettering Cancer Center (MSKCC) by Bercz et al. reported no significant difference in OS between SCRT and LCRT, both in the overall cohort and among patients managed with a watch-and-wait (WW) approach. While 2-year DFS rates were comparable in the WW subgroup (90%), DFS rates in the overall population showed a non-significant trend favoring LCRT (79% vs. 70%, *P* = 0.07), a finding similar to ours; however, our study’s longer follow-up may have enhanced detection of DFS differences (5-year DFS rates: 69.6% LCRT vs. 54.9% SCRT, *P* = 0.07). Also, similar to our findings, the 2-year distant recurrence rates in the whole population were comparable between the two groups (18% LCRT vs 21% SCRT, *P* = 0.6). The MSKCC study found no difference in clinical complete response (cCR) rates. Still, in the WW population LCRT was associated with significantly higher 2-year organ preservation (89% vs. 70%, *P* = 0.005) and a numerically lower local regrowth rate (19% vs. 36%, *P* = 0.07) without any significant difference in the 2-year distant metastasis rates (10% LCRT, vs 6% SCRT, *P* = 0.5), suggesting SCRT may carry a higher risk of local failure in non-operative management, underscoring concerns about SCRT’s durability [24]. Key differences exist between our trial design and the MSKC method. The MSKCC study delivered SCRT as radiotherapy alone using induction or consolidation chemotherapy as part of the TNT approach, whereas our study, though not TNT-based, intensified SCRT with concurrent oxaliplatin and capecitabine, followed by consolidation chemotherapy before surgery. Despite this augmented regimen incorporating concurrent oxaliplatin-based chemotherapy, designed to enhance tumor response while isolating the impact of radiation duration [6, 7], our findings similarly revealed a consistent trend toward improved long-term results with LCRT, suggesting that even chemotherapy-intensified SCRT regimens may not fully reproduce the long-term disease outcomes achieved with LCRT.

In this context, it’s important to note the ongoing ACO/ARO/AIO-18 phase 3 randomized trial (NCT04246684) as a significant step forward in treating LARC. It compares the effectiveness of SCRT, based on the RAPIDO protocol, with conventional CRT regimens, both followed by consolidation chemotherapy and either surgery or a WW approach for patients with cCR. The trial introduces innovations such as strict MRI-based patient selection, the use of proven 5-FU/oxaliplatin-based regimens, and a refined TNT sequencing strategy. The results of this trial are expected to optimize treatment plans while carefully balancing treatment timing, organ preservation, and survival outcomes.

The LCRT approach produced more favorable long-term outcomes in men compared to SCRT. This may be attributed to anatomical and surgical challenges unique to the male pelvis. Research has shown that men undergoing abdominoperineal resection for LARC often experience poorer survival outcomes compared to women, likely due to the narrower dimensions of the male pelvis, which complicates surgical access, affects the oncological outcomes, and increases the risk of postoperative complications. These challenges necessitate a more aggressive adjuvant approach to improve oncological outcomes, such as the higher total radiation dose in LCRT [25, 26]. While recent studies indicate no significant gender differences in radiosensitivity, with similar DNA repair, tumor regression, and lymphocyte infiltration observed in both genders [27], the surgical complexities in men underscore the importance of tailored treatment strategies that address these anatomical differences.

LCRT has been associated with better long-term outcomes compared to SCRT in patients younger than 60, which may be influenced by several factors. First of all, this variation can be attributed to the fact that younger patients tend to have more advanced-stage disease, poorly differentiated tumors, and specific histological subtypes, like mucinous or signet ring cell carcinomas, which increase the risk of early recurrence [28–30]. On the other hand, preclinical studies show that aging weakens the immune system, with fewer naive T-cells, a lower CD8/Treg ratio, and a less favorable tumor environment. Thus, younger patients, with a more functional immune system, tend to respond better to radiotherapy, as it promotes tumor cell death, releases inflammatory signals leading to an anti-tumor response [31–33]. Additionally, fractionated radiotherapy further boosts the immune system by upregulating key proteins that activate cytotoxic T-cells [34]. Therefore, younger patients with more functional immune systems may derive greater benefit from the pro-immunogenic effects of LCRT. In contrast, age-related immune decline in older patients may reduce the efficacy of these mechanisms, resulting in more comparable outcomes between LCRT and SCRT.

Findings revealed a significantly lower DFS with SCRT in cT4 patients compared to LCRT. This may be attributed to the distinct needs of advanced tumors, which likely require more effective downstaging and comprehensive control. Prolonged and fractionated dosing of the LCRT approach might allow more time for tumor shrinkage, which could be crucial for bulky, advanced tumors. Additionally, the higher biologically effective dose of LCRT may offer better control of advanced tumors like T4, whereas SCRT, with its lower biologically effective dose, appears more suitable for less advanced tumors (T2-3). This aligns with previous studies, which found that conventional preoperative radiotherapy is more effective for advanced stages. The higher biologically effective dose of LCRT can produce a more gradual and sustained tumor response, which may be instrumental in reducing residual tumors over time, ultimately improving long-term outcomes like DFS [35].

### Strengths

We mitigated the impact of concurrent chemotherapy by implementing chemotherapy along with the SCRT approach. Additionally, all patients underwent radiotherapy at the same center, following the consistent 3-D protocol, ensuring adherence to the treatment protocol without any concerns about missing treatment due to IMRT.

### Limitations

Not all patients underwent total mesorectal excision due to the long waiting list at the center, and some patients had surgery in another center. Also, we did not administer the TNT or a planned non-operative management approach because it was not the standard protocol of care when the study started. Regarding the long-term toxicity, there was no access to assess these complication results, and the study aimed to evaluate the long-term OS and DFS results.

## Conclusions

Although the preliminary and the updated results of our study did not suggest a significant difference in pCR rates between the two approaches, long-term follow-up results with an added number of patients showed better DFS rates in the LCRT approach, with a significant difference in subgroups including males and patients with a clinical T4 stage and those under the age of 60. Although the study was designed to evaluate an intensified version of SCRT, all trends favored LCRT; thus, a key takeaway from this study is that intensified SCRT still failed to achieve the oncologic outcomes of LCRT. These results suggest that considerable caution is warranted when employing the SCRT approach in patients with LARC. Further studies should be done to compare these two approaches in the TNT setting in lower-risk patients.

## Supplementary Information

Below is the link to the electronic supplementary material.

ESM 1 (DOCX 429 KB)

## Data Availability

The datasets used or analyzed during the current study are available from the corresponding author upon reasonable request.

## Abbreviations

SCRT: Short-course radiotherapy
LCRT: Long-course radiotherapy
OS: Overall survival
DFS: Disease-free survival
LR: Locoregional recurrence
DM: Distant metastasis
LARC: Locally advanced rectal cancer
pCR: Pathological complete response
5 FU: 5-Fluorouracil
TNT: Total neoadjuvant therapy
MRI: Magnetic resonance imaging
EUS: Endoscopic ultrasound
CEA: Carcinoembryonic antigen
3D-CRT: Three-dimensional conformal radiotherapy
SD: Standard deviation
HR: Hazard ratio
TRG: Tumor regression grade
CRC: Colorectal cancer
ITT: Intention-to-treat
IMRT: Intensity-modulated radiation therapy
