# Supplementary material for: Short-course versus long-course neoadjuvant chemoradiotherapy in patients with rectal cancer: long-term results of a randomized controlled trial
Source: Int J Colorectal Dis. 2025 May 14;40(1):118. doi: 10.1007/s00384-025-04901-1 (PMC12078407; doi:10.1007/s00384-025-04901-1)
Supplement: Supplementary file 1 — (DOCX 429 KB) [file 384_2025_4901_MOESM1_ESM.docx]

Supplementary file: Figures S1 to S4


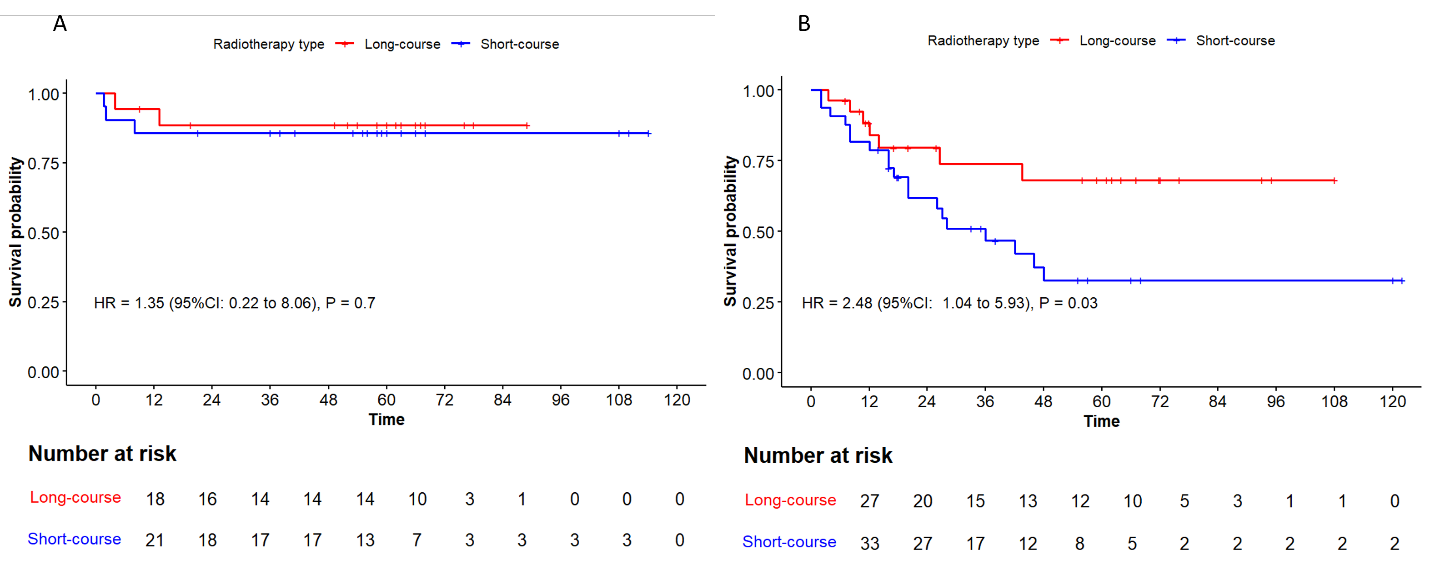


Figure S1. DFS analysis in subgroups based on sex. A) DFS analysis in female patients B) DFS analysis in male patients, p-value by log-rank method.


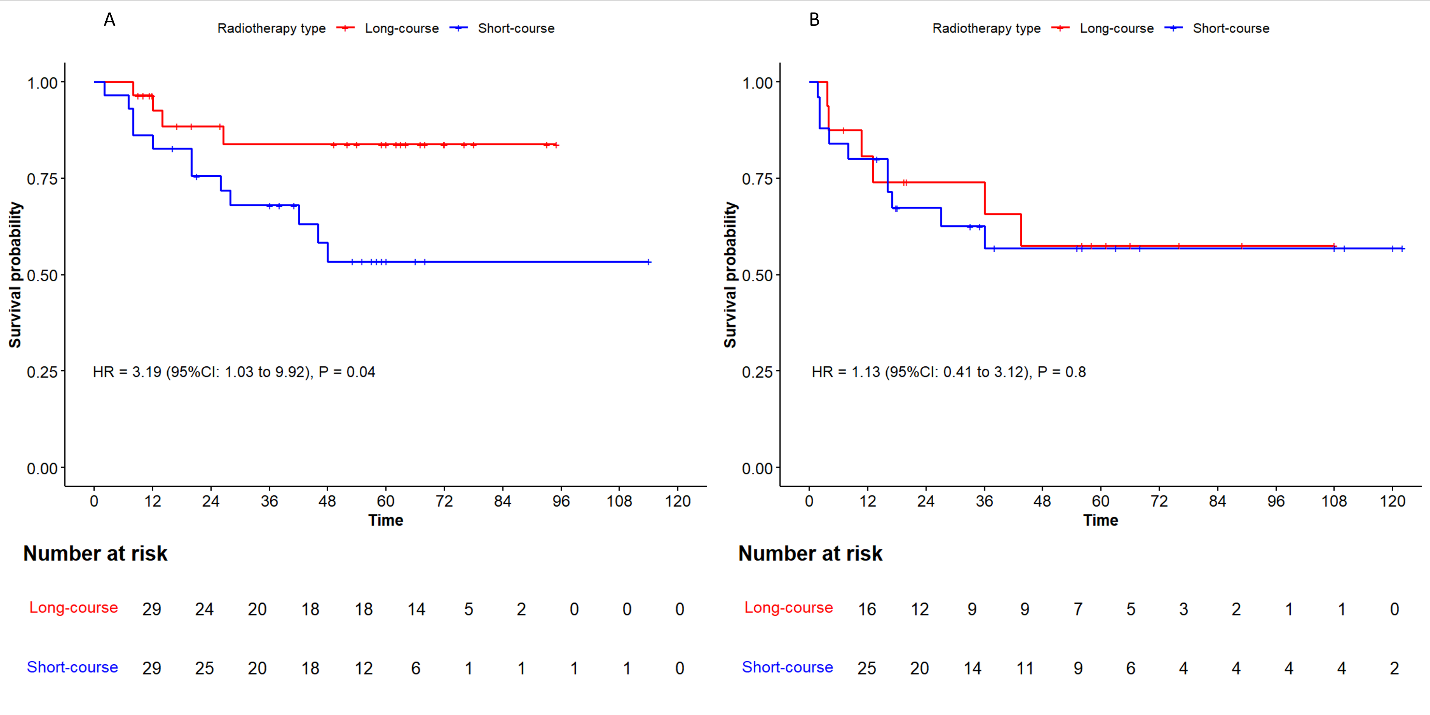


Figure S2. DFS analysis in subgroups based on the age group of patients. A) DFS analysis in ≤ 60 years old patients B) DFS analysis in > 60 years old patients, p-value by log-rank method.


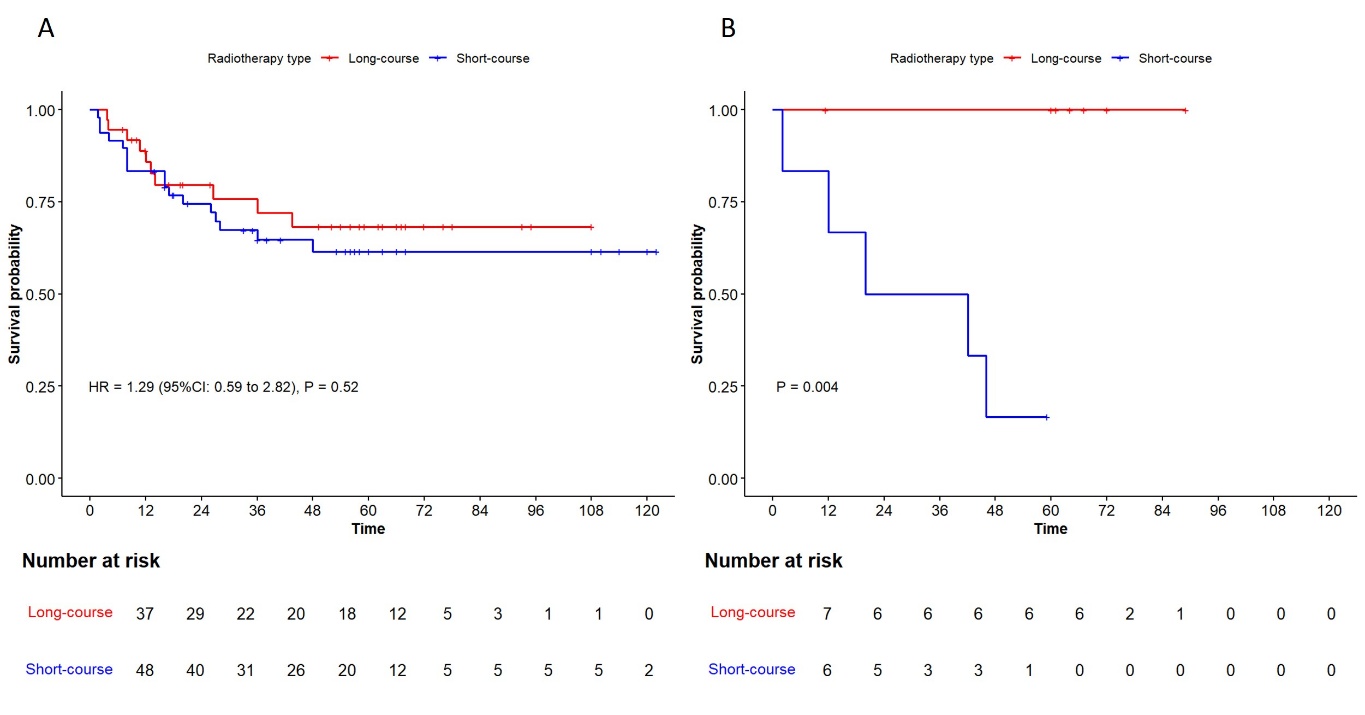


Figure S3. DFS analysis in subgroups based on clinical T status of patients. A) DFS analysis in non-cT4 patients B) DFS analysis in cT4 patients, p-value by log-rank method.


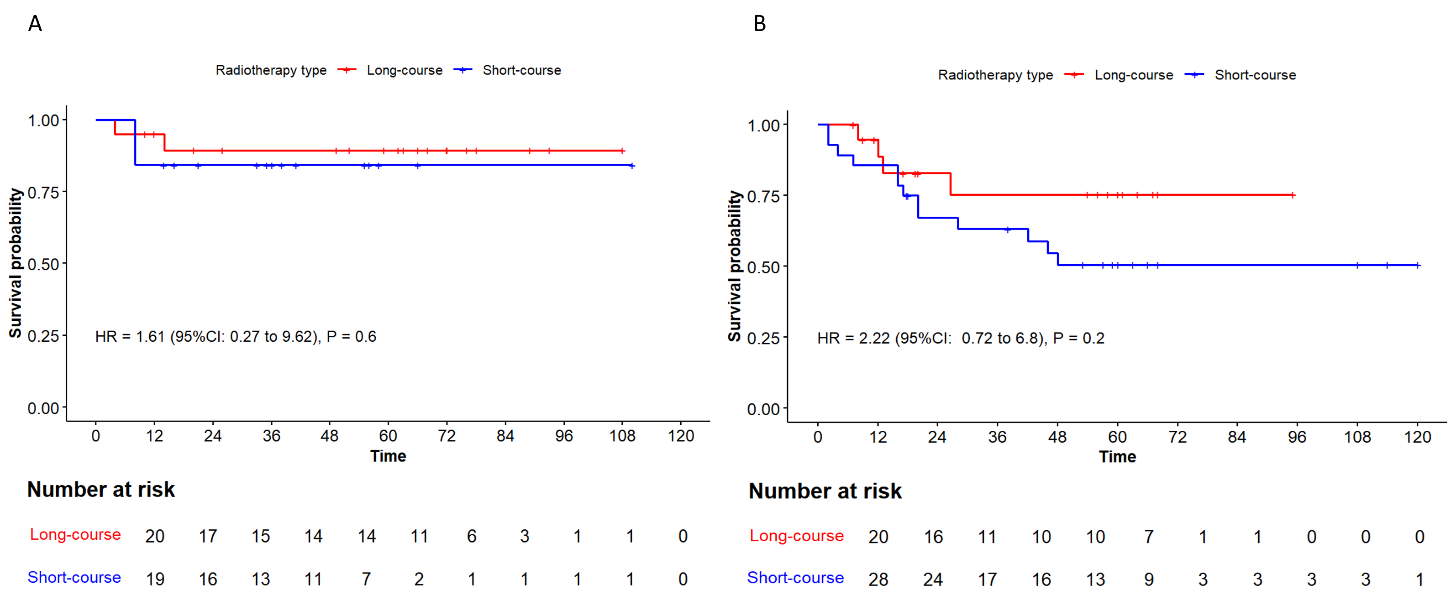


Figure S4. DFS analysis in subgroups based on tumor regression grade (TRG). A) DFS analysis in TRG 0-1 patients B) DFS analysis in TRG 2-3, p-value by log-rank method.
